# Supplementary material for: Higher Rates of Viral Evolution in Chronic Hepatitis B Patients Linked to Predicted T Cell Epitopes
Source: Viruses. 2025 May 8;17(5):684. doi: 10.3390/v17050684 (PMC12115741; doi:10.3390/v17050684)
Supplement: Supplementary file 1 [file viruses-17-00684-s001.zip › viruses-3592459-supplementary.pdf]

Table S1. HLA typing of patients of MHC-1

Patient	Group	HLA-A		HLA-B		HLA-C	
1	Non-treatment	A*23:01:01	A*24:02:01	B*49:01:01	B*55:01:01	C*01:02:01	C*07:01:01
2	Treatment	A*33:03:01	A*11:01:01	B*58:01:01	B*55:02:01	C*01:02:01	
3	Treatment	A*02:01:01	A*33:03:01	B*15:02:01	B*58:01:01	C*03:02:02	
4	Non-treatment	A*03:01:01	A*26:01:01	B*47:01:01	B*55:01:01	C*01:02:01	C*06:02:01
5	Non-treatment	A*11:01:01	A*30:01:01	B*07:02:01	B*35:01:01	C*07:02:01	
6	Treatment	A*02:06:01	A*29:01:01	B*15:02:01	Undetermined	C*15:05:02	
7	Non-treatment	A*02:01:01	A*24:02:40	B*55:02:01	B*46:01:01	C*04:01:01	C*01:02:01
9	Treatment	A*11:01:01	A*33:03:01	B*44:03:01	B*15:01:01	C*14:03:01	C*04:01:01
10	Treatment	A*24:02:01		B*51:01:01	B*52:01:01	C*01:02:01	C*12:02:02
12	Treatment	A*24:02:01		B*40:01:02		C*07:02:01	
14	Non-treatment	A*29:02:01	A*30:01:01	B*41:01:01	B*07:02:01	C*15:02:02	C*17:01:01
15	Treatment	A*33:03:01		B*44:03:02	B*58:01:01	Undetermined	
18	Treatment	A*02:06:01	A*11:01:01	B*38:02:01	Undetermined	C*04:01:01	
19	Non-treatment	A*34:02:01		B*44:03:01	B*07:02:01	C*04:01:01	C*07:02:01
20	Treatment	A*03:01:01	A*24:02:01	B*51:01:01	B*52:01:01	C*12:02:02	C*14:02:01
21	Treatment	A*03:01:01	A*24:02:01	B*18:01:01	B*15:01:01	C*07:01:01	
22	Treatment	A*11:01:01	A*32:01:01	B*18:01:01	B*55:01:01	C*01:02:01	C*12:03:01
24	Treatment	A*11:01:01		B*15:02:01	B*40:01:02	C*03:04:01	C*08:01:01
25	Treatment	A*02:01:01	A*32:01:01	B*51:01:01	B*67:01:01	C*14:02:01	

Table S2. Comparison of predicted CD8+ T cell epitopes across the three different time points (A-C) for all patients

Patient	Group	HLA restriction	Protein	Start	Finish	Length	Time point A	Total score	Time point B	Total score	Time point C	Total score
1	Non-treatment	HLA-A*23:01	C	109	118	10	MFGRQTVVEF	0.51	TFGRQTVVEY	<0	TFGRQTIVEY	<0
		HLA-A*24:02	C	109	118	10	MFGRQTVVEF	0.37	TFGRQTVVEY	<0	TFGRQTIVEY	<0
		HLA-A*23:01	C	116	125	10	VEFLVSFGVW	<0	VEYLVSFGVW	<0	IEYLVSFGVW	0.01
		HLA-A*23:01	C	101	110	10	LWFHISCLMF	1.28	LWFHISCLTF	1.64	LWFHISCLTF	1.64
		HLA-A*24:02	C	101	110	10	LWFHISCLMF	0.70	LWFHISCLTF	1.23	LWFHISCLTF	1.23
		HLA-A*23:01	C	102	110	9	WFHISCLMF	0.70	WFHISCLTF	0.83	WFHISCLTF	0.83
		HLA-A*23:01	PreS	374	382	9	PFLALLPIF	0.46	PFLALLPIF	0.46	PFLPLLPIF	0.79
		HLA-A*23:01	PreS	373	382	10	SPFLALLPIF	0.54	SPFLALLPIF	0.54	SPFLPLLPIF	0.82
2	Treatment	HLA-A*33:03	C	125	133	9	WIRTPPAYR	0.47	WIRTPPGYR	<0	WIRTPPPYR	0.16
		HLA-B*58:01	Polymerase	164	175	12	RSASFCGSPYSW	1.07	RSASFYGSPYSW	1.11	RSASFYGSPYSW	1.11
		HLA-B*58:01	Polymerase	163	175	13	TRSASFCGSPYSW	0.66	TRSASFYGSPYSW	0.70	TRSASFYGSPYSW	0.70
		HLA-B*58:01	Polymerase	165	175	11	SASFCGSPYSW	0.69	SASFYGSPYSW	0.67	SASFYGSPYSW	0.67
		HLA-B*58:01	Polymerase	395	404	10	FSRGNTRVSW	0.12	FSRGNTRVSW	0.12	FSRGSTHVSW	0.43
		HLA-B*58:01	Polymerase	398	407	10	GNTRVSWPKF	<0	GNTRVSWPKF	<0	GSTHVSWPKF	0.60
3	Treatment	HLA-B*58:01	Polymerase	558	567	10	KSVQHLEALY	1.26	KSVQHLESLY	1.23	KSVQHLEALY	1.26
		HLA-B*15:02	Polymerase	565	573	9	ALYTAVTNF	0.28	SLYAAVTNF	0.77	ALYTAVTNF	0.28
		HLA-A*02:01	Polymerase	567	575	9	YTAVTNFLL	0.14	YAAVTNFLL	0.18	YTAVTNFLL	0.14
4	Non-treatment	HLA-A*03:01	PreS	352	361	10	MMWYWGPSLY	1.72	**NO SEQ**		MMWYWGPSLC	<0
5	Non-treatmeant	HLA-C*07:02	C	132	140	9	**NO SEQ**		YRPVNAPIL	0.59	YRPPNAPIL	0.84
		HLA-B*35:01	PreS	52	61	10			FPGHQLDPAF	1.15	FPSHQLDPAF	1.47
		HLA-B*35:01	PreS	327	335	9			IPALGTSMF	1.24	IPAQGTSMF	1.24
		HLA-B*07:02	PreS	327	335	9			IPALGTSMF	0.51	IPAQGTSMF	0.40
		HLA-B*07:02	C	133	143	11			RPVNAPILSTL	0.83	RPPNAPILSTL	0.27
6	Treatment	HLA-B*15:02	PreS	145	154	10	QAMQWNSSTF	0.69	QAMQWNSSTF	0.69	QAIQWNSSTF	<0
		HLA-B*15:02	PreS	146	154	9	AMQWNSSTF	0.58	AMQWNSSTF	0.58	AIQWNSSTF	<0
7	Non-treatmeant	HLA-A*24:02	PreS	249	257	9	MYLRRFIIF	1.42	MCLRRFIIF	<0	MCLRRFIIF	<0
		HLA-A*24:02	PreS	248	257	10	WMYLRRFIIF	0.64	WMCLRRFIIF	<0	WMCLRRFIIF	<0
		HLA-A*24:02	PreS	247	256	10	RWMYLRRFII	<0	RWMCLRRFII	0.14	RWMCLRRFII	0.14
		HLA-A*02:01	PreS	250	258	9	YLRRFIIFL	0.55	CLRRFIIFL	<0	CLRRFIIFL	<0
		HLA-A*02:01	X	92	100	9	VLHKRTLGL	<0	VLYKRTLGL	0.18	VLYKRTLGL	0.18
		HLA-A*24:02	C	101	110	10	LWFHISCLTF	1.24	LWFHISCLMF	0.71	LWFHISCLTF	1.24

		HLA-A*02:01	PreS	371	379	9	MIWYWGPSL	0.20	MIWYWGPSL	0.20	MMWYWGPSL	1.19
		HLA-A*02:01	PreS	370	379	10	WMIWYWGPSL	0.48	WMIWYWGPSL	0.48	WMMWYWGPSL	0.83
		HLA-A*24:02	PreS	364	373	10	VWLSVIWMIW	0.18	VWLSVIWMIW		VWLSVIWMMW	0.15
9	Treatment	**No changes**										
10	Treatment	**No changes**										
12	Treatment	HLA-A*24:02	C	101	110	10	LWFHISCLSF	0.88	LWFHISCLTF	1.25	LWFHISCLSF	0.88
		HLA-C*07:02	C	132	140	9	YRPQNAPIL	0.62	YRPPNAPIL	0.84	YRPPNAPIL	0.84
		HLA-A*24:02	Polymerase	62	70	9	LYSSTVPCF	1.13	LYSSTVPCF	1.13	LYSSTVPRF	1.29
		HLA-A*24:02	Polymerase	4	13	10	SYQHFRKLLL	<0	SYQHFRKLLL	<0	SYQRFRKLLL	0.15
		HLA-C*07:02	Polymerase	6	14	9	QHFRKLLLL	<0	QHFRKLLLL	<0	QRFRKLLLL	0.07
14	Non-treatment	HLA-A*29:02	PreS	360	369	10	TMWYWGPNLY	2.18	MMWYWGPNLY	2.45	TMWYWGPNLY	2.18
		HLA-A*29:02	PreS	359	369	11	WTMWYWGPNLY	1.52	WMMWYWGPNLY	1.90	WTMWYWGPNLY	1.52
		HLA-A*29:02	PreS	358	369	12	IWTMWYWGPNLY	1.43	IWMMWYWGPNLY	1.89	IWTMWYWGPNLY	1.43
		HLA-A*29:02	PreS	356	369	14	SVIWTMWYWGPNLY	1.42	LAIWMMWYWGPNLY	1.61	SVIWTMWYWGPNLY	1.42
		HLA-A*29:02	PreS	354	363	10	WLSVIWTMWY	0.68	WLLAIWMMWY	0.60	WLSVIWTMWY	0.68
		HLA-A*29:02	C	109	118	10	TFGRDTVLEY	0.79	TFGRQTVVEY	<0	TFGRQTVVEY	<0
		HLA-A*29:02	C	108	118	11	LTFGRDTVLEY	0.69	LTFGRQTVVEY	<0	LTFGRQTVVEY	<0
		HLA-B*07:02	Polymerase	71	79	9	NPHWKTPSF	0.59	NPHWKTPYF	<0	NPHWKTPYF	<0
		HLA-B*07:02	Polymerase	70	79	10	FNPHWKTPSF	0.47	FNPHWKTPYF	<0	FNPHWKTPYF	<0
		HLA-B*07:02	X	28	37	10	RPFSGPLGTL	0.86	RPFSGPLGTL	0.86	RPVSGPLGTL	0.90
15	Treatment	HLA-A*33:03	Polymerase	136	145	10	HTVNHYFQAR	0.57	HTVNHYFQTR	0.55	HTVNHYFQTR	0.55
		HLA-A*33:03	Polymerase	137	145	9	TVNHYFQAR	0.39	TVNHYFQTR	0.36	TVNHYFQTR	0.36
		HLA-B*58:01	Polymerase	143	152	10	QARHYLHTLW	0.29	QTRHYLHTLW	0.29	QTRHYLHTLW	0.29
18	Treatment	**No changes**										
19	Non-treatment	HLA-C*07:02	Polymerase	791	799	9	YRPLLRLPF	0.50	SRPLLRLPF	<0	SRPLLRLPF	<0
		HLA-B*07:02	Polymerase	707	716	10	RVRGTFVAPL	0.22	RMRGTFVAPL	<0	RMRGTFVAPL	<0
		HLA-B*44:03	X	79	88	10	METTVNAHQL	<0	METTVNAHQF	0.52	METTVNAHQF	0.52
20	Treatment	**No changes**									**No Seq**	
21	Treatment	**No changes**										
22	Treatment	HLA-C*12:03	C	80	88	9	ISRDLVVSY	1.07	ISRDLVVSY	1.07	TSRDLVVSY	0.80
24	Treatment	**No changes**										
25	Treatment	HLA-C*14:02	Polymerase	140	148	9	HYFQTRHYL	1.53	HYFKTRHYL	1.41	HYFKTRHYL	1.41
		HLA-A*32:01	Polymerase	143	151	9	QTRHYLHTL	<0	KTRHYLHTL	0.28	KTRHYLHTL	0.28
		HLA-A*32:01	Polymerase	143	152	10	QTRHYLHTLW	<0	KTRHYLHTLW	0.08	KTRHYLHTLW	0.08
		HLA-C*14:02	Polymerase	139	148	10	NHYFQTRHYL	0.02	NHYFKTRHYL	<0	NHYFKTRHYL	<0

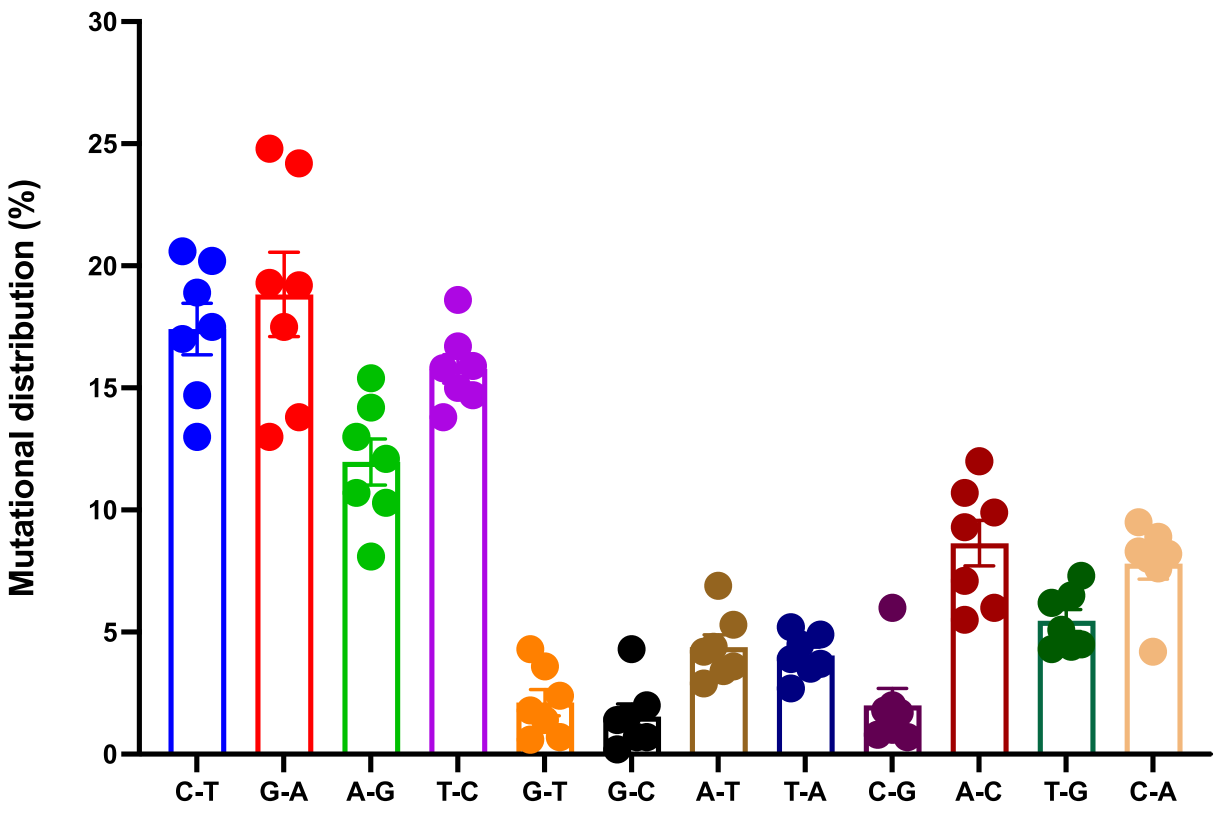

Figure S1. Distribution and frequency of total SNPs transitions and transversion. This was performed for 7 patients with sufficient data. Each transition or transversion are represented as a mean frequency (%) of total SNPs with error bars as SEM and individual patients shown as dot.
